# Supplementary material for: Polygynous marriage union among Ghanaian Christian women: Socio-demographic predictors
Source: PLoS One. 2023 Apr 27;18(4):e0275764. doi: 10.1371/journal.pone.0275764 (PMC10138464; doi:10.1371/journal.pone.0275764)
Supplement: Attachment — Submitted filename: PONE-D-22-26419_reviewer-1.pdf [file pone.0275764.s002.pdf]

PLOS ONE

Abdul Rauf Alhassan

--Manuscript Draft--

Manuscript Number:	PONE-D-22-26419
Article Type:	Research Article
Full Title:	Abdul Rauf Alhassan
Short Title:	Polygyny in Christianity
Corresponding Author:	Abdul Rauf Alhassan, MPH<br>Tamale Teaching Hospital<br>Tamale, Northern Region GHANA
Keywords:	Christianity, Determinants, Polygamy, Polygyny, Women
Abstract:	Introduction: Polygamy has declined in the last decade, but it is still prevalent in West African nations including Ghana even with the arrival of Christianity and colonists, which came to be recognized as a form of slavery that needed to be abolished. Aim: To analyze the determinants of polygyny among married Christian women in Ghana. Methods: Ghana Maternal Health Survey data was used for this study to do an analytic cross-section study. Data analysis was done using SPSS version 20. The association between dependent and independent variables was explored using chi-square and logistic regression. Statistical significance was set at $p < 0.05$. Results: The prevalence of Ghanaian Christian women's involvement in polygyny marriage union was 12.2%, the prevalence was higher (15.0%) among women of Anglican denomination, catholic denomination (13.9%), and the lowest (8.4%) prevalence recorded among those of Methodist denominations. The predictor factors identified include the age of the woman, history of education, type of place of residence, region, ethnicity, early sex initiation, and history of multiple unions. Conclusion: The prevalence of polygyny in this present study is high given the strict position the Christian religion has against polygyny. This study recommends that the pros and cons of polygyny are objectively looked at from a scientific point rather than a religious point of view.
Order of Authors:	Abdul Rauf Alhassan, MPH
Additional Information:	
**Question**	**Response**
**Financial Disclosure**<br><br>Enter a financial disclosure statement that describes the sources of funding for the work included in this submission. Review the submission guidelines for detailed requirements. View published research articles from *PLOS ONE* for specific examples.<br><br>This statement is required for submission and **will appear in the published article** if the submission is accepted. Please make sure it is accurate.	Funding for this study was completed by Ghana Organization for Maternal and Child Health (GOMaCH). The funder had no role in study design, data collection and analysis, or preparation of the manuscript.

Powered by Editorial Manager® and ProduXion Manager® from Aries Systems Corporation

**Unfunded studies**<br>Enter: *The author(s) received no specific funding for this work.*<br><br>**Funded studies**<br>Enter a statement with the following details:<br>• Initials of the authors who received each award<br>• Grant numbers awarded to each author<br>• The full name of each funder<br>• URL of each funder website<br>• Did the sponsors or funders play any role in the study design, data collection and analysis, decision to publish, or preparation of the manuscript?<br>• **NO** - Include this sentence at the end of your statement: *The funders had no role in study design, data collection and analysis, decision to publish, or preparation of the manuscript.*<br>• **YES** - Specify the role(s) played.<br><br>* typeset	
**Competing Interests**<br><br>Use the instructions below to enter a competing interest statement for this submission. On behalf of all authors, disclose any competing interests that could be perceived to bias this work—acknowledging all financial support and any other relevant financial or non-financial competing interests.<br><br>This statement is required for submission and **will appear in the published article** if the submission is accepted. Please make sure it is accurate and that any funding sources listed in your Funding Information later in the submission form are also declared in your Financial Disclosure statement.<br><br>View published research articles from *PLOS ONE* for specific examples.	The authors have declared that no competing interests exist.

**NO authors have competing interests**<br><br>Enter: *The authors have declared that no competing interests exist.*<br><br>**Authors with competing interests**<br><br>Enter competing interest details beginning with this statement:<br><br>*I have read the journal's policy and the authors of this manuscript have the following competing interests: [insert competing interests here]*<br><br>* typeset	
**Ethics Statement**<br><br>Enter an ethics statement for this submission. This statement is required if the study involved:<br><br>• Human participants<br>• Human specimens or tissue<br>• Vertebrate animals or cephalopods<br>• Vertebrate embryos or tissues<br>• Field research<br><br>Write "N/A" if the submission does not require an ethics statement.<br><br>General guidance is provided below. Consult the submission guidelines for detailed instructions. **Make sure that all information entered here is included in the Methods section of the manuscript.**	The ICF Institutional Review Board approved the protocol for the 2017 GMHS. Meanwhile, ethical approval was not required for this study because it involved a secondary analysis of a dataset without exposing the respondents' and their households' identities. Nonetheless, permission to use the datasets in this study was obtained from ICF via the DHS program, and the data terms followed.

**Format for specific study types**<br><br>**Human Subject Research (involving human participants and/or tissue)**<br>• Give the name of the institutional review board or ethics committee that approved the study<br>• Include the approval number and/or a statement indicating approval of this research<br>• Indicate the form of consent obtained (written/oral) or the reason that consent was not obtained (e.g. the data were analyzed anonymously)<br><br>**Animal Research (involving vertebrate animals, embryos or tissues)**<br>• Provide the name of the Institutional Animal Care and Use Committee (IACUC) or other relevant ethics board that reviewed the study protocol, and indicate whether they approved this research or granted a formal waiver of ethical approval<br>• Include an approval number if one was obtained<br>• If the study involved *non-human primates*, add *additional details* about animal welfare and steps taken to ameliorate suffering<br>• If anesthesia, euthanasia, or any kind of animal sacrifice is part of the study, include briefly which substances and/or methods were applied<br><br>**Field Research**<br><br>Include the following details if this study involves the collection of plant, animal, or other materials from a natural setting:<br>• Field permit number<br>• Name of the institution or relevant body that granted permission	
**Data Availability**<br><br>Authors are required to make all data underlying the findings described fully available, without restriction, and from the time of publication. PLOS allows rare exceptions to address legal and ethical concerns. See the PLOS Data Policy and FAQ for detailed information.	Yes - all data are fully available without restriction

A Data Availability Statement describing where the data can be found is required at submission. Your answers to this question constitute the Data Availability Statement and **will be published in the article**, if accepted.<br><br>**Important:** Stating 'data available on request from the author' is not sufficient. If your data are only available upon request, select 'No' for the first question and explain your exceptional situation in the text box.<br><br>Do the authors confirm that all data underlying the findings described in their manuscript are fully available without restriction?	
**Describe where the data may be found in full sentences. If you are copying our sample text, replace any instances of XXX with the appropriate details.**<br><br>• If the data are **held or will be held in a public repository**, include URLs, accession numbers or DOIs. If this information will only be available after acceptance, indicate this by ticking the box below. For example: *All XXX files are available from the XXX database (accession number(s) XXX, XXX.).*<br>• If the data are all contained **within the manuscript and/or Supporting Information files**, enter the following: *All relevant data are within the manuscript and its Supporting Information files.*<br>• If neither of these applies but you are able to provide **details of access elsewhere**, with or without limitations, please do so. For example:<br><br>*Data cannot be shared publicly because of [XXX]. Data are available from the XXX Institutional Data Access / Ethics Committee (contact via XXX) for researchers who meet the criteria for access to confidential data.*<br><br>*The data underlying the results presented in the study are available from (include the name of the third party*	All dataset related to the findings of this study is available online at www.dhsprogram.com.

and contact information or URL).<br>• This text is appropriate if the data are owned by a third party and authors do not have permission to share the data.<br><br>* typeset	
Additional data availability information:	Tick here if your circumstances are not covered by the questions above and you need the journal’s help to make your data available.

Powered by Editorial Manager® and ProduXion Manager® from Aries Systems Corporation

Manuscript Click here to access/download;Manuscript;Manuscript.docx

Polygynous Marriage Union among Ghanaian Christian Women: Socio-demographic Predictors

Abdul Rauf Alhassan[1]; HasbiRC[2]

1. Department of Surgery, Tamale Teaching Hospital, P.O. Box TL 16; Tamale-Ghana
2. Hasbi Research Consultancy, Tamale, Ghana

Correspondence should be addressed to Abdul Rauf Alhassan: Alhassana84@yahoo.com or abdul-rauf.alhassan@tth.gov.gh or ORCID ID: 0000-0003-2252-2646)

Running Title: Polygyny in Christianity.

Abstract

Introduction: Polygamy has declined in the last decade, but it is still prevalent in West African nations including Ghana even with the arrival of Christianity and colonists, which came to be recognized as a form of slavery that needed to be abolished. **Aim:** To analyze the determinants of polygyny among married Christian women in Ghana. **Methods:** Ghana Maternal Health Survey data was used for this study to do an analytic cross-section study. Data analysis was done using SPSS version 20. The association between dependent and independent variables was explored using chi-square and logistic regression. Statistical significance was set at $p < 0.05$. **Results:** The prevalence of Ghanaian Christian women's involvement in polygyny marriage union was 12.2%, the prevalence was higher (15.0%) among women of Anglican denomination, catholic denomination (13.9%), and the lowest (8.4%) prevalence recorded among those of Methodist denominations. The predictor factors identified include the age of the woman, history of education, type of place of residence, region, ethnicity, early sex initiation, and history of multiple unions. **Conclusion:** The prevalence of polygyny in this present study is high given the strict position the Christian religion has against polygyny. This study recommends that the pros and cons of polygyny are objectively looked at from a scientific point rather than a religious point of view.

Keywords: Christianity, Determinants, Polygamy, Polygyny, Women

Introduction

Polygamy is a type of marriage in which several spouses are involved. It can happen as polygyny (when a man has multiple wives at the same time). It can also be polyandry (when a woman has multiple husbands at the same time), or polygynandry (concurrent marriage of two or more wives to two or more husbands) (Al-Krenawi & Graham, 2009; Al-Krenawi & Slonim-Nevo, 2008; Al-Krenawi, 2001; Elbedour, Onwuegbuzie, Caridine, & Abu-Saad, 2002).

The most common form of polygamy is polygyny. More than 80% of preindustrial societies had it (Hassouneh-Phillips, 2001). Even though the global prevalence of polygyny is low, more than a third of the world's population lives in a community that allows it (Thobejane & Flora, 2014). Polygyny has been practiced by various cultures throughout the world for many centuries. In most African countries, it has been an essential component of family law. However, with the arrival of Christianity and colonists, it came to be recognized as a form of slavery that needed abolishment. As a result, its prevalence has been steadily decreasing for decades. Despite this, it is still more prevalent in Sub-Saharan Africa (SSA) than anywhere else (Mwambene, 2017). The 'polygyny belt,' which stretches from Senegal in West Africa to Tanzania in East Africa, has the highest prevalence of polygyny in Africa (Jacoby, 1995). According to Demographic and Health Survey (DHS) data, 11 percent, 27 percent, and 53 percent of marriages in Zimbabwe, Ivory Coast, and Guinea were polygynous (Bove & Valeggia, 2009). According to another DHS report, polygyny accounts for 25% of all marriages in the Democratic Republic of the Congo (DRC), 47% in Sierra Leone, and 53% in The Gambia (Trócaire, 2017).

In Ghana, a study done using nationally representative data from 2017/2018 Ghana Multiple Indicator Cluster Survey revealed the prevalence of polygyny to be 21.6% (Adewale, Dey, Ansah, Duah, & Agbadi, 2021).

Demographic factors such as high infant and child mortality, high male mortality and outmigration, and potentially lethal male activities such as hunting and military combat contribute to an excess supply of women and a scarcity of men, which can promote polygyny (Fulas, 2018; Gibson & Mace, 2007). Polygyny has become more common due to religion, particularly Mormonism and Islam. Age, place of residence, and household wealth all influence the prevalence of polygyny (Okorje, 1994; Strassmann, 1997). Other major factors influencing polygyny acceptance include culture and tradition. Polygyny is recommended as the solution to infertility and menopause in many African cultures. Polygyny increases the number of children available for domestic work, farming, and cattle herding in agricultural societies (Elbedour, Onwuegbuzie, Caridine, & Abu-Saad, 2002; Mwambene, 2017; Slonim-nevo & Al-Krenawi, 2006). Polygyny is associated with an increased risk of infant and child mortality (Smith-Greenaway & Trinitapoli, 2014; Omariba & Boyle, 2007). Children from polygynous families have poor health and die from malnutrition and HIV/AIDS (Hadley, 2005; Lawson & Gibson, 2018). Many studies have identified polygyny as one of the factors that influence early marriage, domestic violence, harmful traditional practices, and high fertility (Ahinkorah, 2021; Gaffney-Rhys, 2012).

Polygamy has declined in the last decade, but it is still prevalent in West Africa. This ancient practice is encouraged by customary law and/or religious practices. Polygamy is also recognized and governed by civil law across most West African countries, allowing a man to marry up to four women in certain situations, including the financial capacity to support multiple wives and families (SWAC/OECD, 2019). In most cases, a polygamous union is limited to two women per couple. Six Countries In Africa (Benin, Cabo Verde, Côte d'Ivoire, Ghana, Guinea, and Nigeria) have civil codes that formally prohibit polygamy, but the laws are poorly applied. Other nations,

such as Burkina Faso and Togo, recognize polygamous unions under contemporary civil law, but only permit couples or men to be involved (SWAC/OECD, 2019).

With the arrival of Christianity and colonists, it came to be recognized as a form of slavery that needed to be abolished (Mwambene, 2017). Polygamy has been a source of consternation for Christian missionaries. In the past, Christian mission institutions in a broad sense have been unable to reach an agreement on how to address this issue. In the highlands of New Guinea, for example, Roman Catholic and Lutheran missions condemn polygamy, refuse to baptize members of polygamous marriages, and demand that polygamous marriages be dissolved. They regard polygamy as a sin. Baptist and Methodist missions, but on the other hand, baptize those who entered a polygamous marriage before hearing the Gospel or, more particularly, before actually deciding to accept Christ. They do not regard polygamy to be a sin, but they believe it is not God's ideal. The view of all these missions is from the Bible (Holst, 1967). This has motivated this current study to conduct a multilevel analysis of determinants of polygyny among married Christian women in Ghana.

Materials and Methods

This study is based on data from the 2017 Ghana Maternal Health Survey in an analytic cross-sectional survey design (GMHS). The Ghana Statistical Service (GSS) with technical help from the ICF’s Demographic and Health Survey (DHS) program carried out the 2017 GMHS. The sampling frame was derived from Ghana's 2010 Population and Housing Census (PHC). Women between the ages of 15 and 49 years who were permanent residents of selected households or guests who stayed in selected households the night before the survey were eligible to participate. A multistage stratified cluster sampling technique was used to select study areas and households.

The final report contains specifics about the survey procedures and questionnaires used (Ghana Statistical Service, 2018).

The study included all survey's currently married Christian women participants (6393), and the study's primary dependent variable was polygyny marriage union. Demographic characteristics, premature sexual initiation, early marriage, and several union experiences were among the independent variables.

Statistical Analysis

SPSS Statistics for Windows, version 20.0, used for statistical analysis (IBM SPSS Statistics for Windows, Version 20.0. Armonk, NY: IBM Corp). Tables and figures are used to present the results of categorical variables using frequencies and percentages. The mean and standard deviation of continuous variables are used to represent the results. The chi-square test was used to determine the relationship between the dependent and independent variables. A binary logistics regression model was used to identify predictor variables of polygyny marriage union for factors that showed a significant association at the bivariate level of analysis using chi-square. A p-value of less than 0.05 was used to define statistical significance.

Ethical consideration

The ICF Institutional Review Board approved the protocol for the 2017 GMHS. Meanwhile, ethical approval was not required for this study because it involved a secondary analysis of a dataset without exposing the respondents' and their households' identities. Nonetheless, permission to use the datasets in this study was obtained from ICF via the DHS program, and the data terms followed.

Results

Demographic characteristics of study participants

The majority (70.2%) of the study participants were 30 years and above, and about 53.7% of them were residing in rural areas. The Christian domination that dominated the (49.1%) study participants was a Pentecostal or charismatic group and the region with dominant (16.4%) representation was the Upper East region. Moreover, the ethnic group with the majority (40.0%) representation was Akan. About half (50.4%) had early sexual initiation, 29.7% were into child marriage, and 14.8% had had more than one union experience.

Prevalence of Christian women involved in a polygyny marriage

The prevalence of Ghanaian Christian women's involvement in polygyny marriage union was 12.2%, the prevalence was higher (15.0%) among women of Anglican denomination, catholic denomination (13.9%), and the lowest (8.4%) prevalence recorded among those of Methodist denomination (Figure 1). The majority (46.9%) of the women in the polygyny marriage union were first wives in terms of rank, and then 45.6% of them were second wives.

Factors associated with Christian women involved in a polygyny marriage

Chi-square analysis revealed that all independent variables included in the study had a significant association with Christian women involved in polygyny marriage (Table 2). These variables were further modeled using a binary logistics regression model to identify predictor variables of Christian women involved in polygyny marriage.

Firstly, the age of the women predicted they're involved in polygyny marriage, those aged 30 years and above were 540% more likely to involve in polygyny marriage union compared to those aged 15-19 years (AOR = 6.4, 95%, C.I = 2.7-14.9). Secondly, those without a history of education were 70% more likely to engage in polygyny marriage union compared to those with

(AOR = 1.7, 95% CI= 1.4-2.0). In addition, those of rural residence was 120% more likely to engage in polygyny marriage compared to those of urban residence (AOR = 2.2, 95% CI= 1.7-2.7). Northern regions were 70% more likely to engage in polygyny marriage unions compared to those of Western regions (AOR = 1.7, 95% CI=1.1-2.6). Again, ethnicity also predicted women's involvement in polygyny marriage unions; those of Ewe tribes were 70% more likely to engage in polygyny marriage unions when compared to those of Akan tribes (AOR =1.7, 95%, CI=1.1-2.5). In addition, those of the Mole-Dagbani tribe were 200% more likely to engage in polygyny marriage unions when compared to those of Akan tribes (AOR =3.0, 95%, CI=2.1-4.3). Again, those of the Gurma tribe were 270% more likely to engage in polygyny marriage unions when compared to those of the Akan tribe (AOR =3.7, 95% CI=2.5-5.5). Furthermore, the Christian denomination the women belong to predicted their involvement in the polygyny marriage union. Those of Methodist denominations were 90% more likely to engage in polygyny marriage union compared to those of Catholic denominations (AOR = 1.9, 95% CI = 1.2-3.0). In addition, those of Presbyterian denominations were 70% more likely to engage in polygyny marriage union compared to those of Catholic denominations (AOR = 1.7, 95% CI = 1.1-2.6). Again, those of Pentecostal/charismatic denominations were 40% more likely to engage in polygyny marriage union compared to those of Catholic denominations (AOR = 1.4, 95% CI = 1.2-1.8). Nevertheless, women with a history of the earlier sexual debut were 50% more likely to engage in polygyny marriage union compared to those without (AOR = 1.5, 95% CI=1.2-1.8). Lastly, women with a history of in-union more than once were 80% more likely to engage in polygyny marriage union compared to those without (AOR = 1.8, 95% CI=1.5-2.2) (Table 3).

Discussion

According to Demographic and Health Survey (DHS) data, 11 percent, 27 percent, and 53 percent of marriages in Zimbabwe, Ivory Coast, and Guinea were polygynous (Bove & Valeggia, 2009). According to another DHS report, polygyny accounts for 25% of all marriages in the Democratic Republic of the Congo (DRC), 47% in Sierra Leone, and 53% in The Gambia (Trócaire, 2017). In Ghana, the prevalence is 21.6% (Adewale, Dey, Ansah, Duah, & Agbadi, 2021). With the arrival of Christianity and colonists, it came to be recognized as a form of slavery that needed abolishment. As a result, its prevalence has been steadily decreasing for decades. Despite this, it is still more prevalent in Sub-Saharan Africa (SSA) than anywhere else (Mwambene, 2017). This is evidenced in this present study, as polygyny was recorded to be 12.2% among Christians compared to 21.6% for the whole Ghana population, which included Muslims and traditional believers who accept the practice (Adewale, Dey, Ansah, Duah, & Agbadi, 2021). In most cases, a polygamous union is limited to two women per couple (SWAC/OECD, 2019). This explains the findings of this current study in which the majority were first wives and second wives in terms of wife's rank.

In the past, Roman Catholic and Lutheran missions condemn polygamy, refuse to baptize members of polygamous marriages, and demanded that polygamous marriages be dissolved. They regard polygamy as a sin. Baptist and Methodist missions, but on the other hand, baptize those who entered a polygamous marriage before hearing the Gospel or, more particularly, before actually deciding to accept Christ. They do not regard polygamy to be a sin, but they believe it is not God's ideal. The view of all these missions is from the Bible (Holst, 1967). However, this present study revealed a higher prevalence among women of Anglican and catholic denominations, and the lowest prevalence recorded among those of Methodist denominations. Meanwhile, further multiple variable analysis revealed that those of Methodist

denominations were more likely to engage in polygyny marriage union compared to those of Catholic denominations. In addition, those of Presbyterian denominations were more likely to engage in polygyny marriage union compared to those of Catholic denominations. Again, those of Pentecostal/charismatic denominations were more likely to engage in polygyny marriage union compared to those of Catholic denominations.

Age may influence the prevalence of polygyny (Okorje, 1994; Strassmann, 1997). Many studies have identified polygyny as one of the factors that influence early marriage (Ahinkorah, 2021; Gaffney-Rhys, 2012). However, in this present study, child marriage did not predict polygyny among women, this further explained with those aged 30 years, and above are more likely, to involve in a polygyny marriage union compared to those aged 15-19 years. This study finding is not different from the results of all demographic and Health Surveys of sub-Saharan African countries conducted since 2000 for 22 countries in which older age was positively associated with polygyny in each country result (CPS/MS, 2007).

Place of residence, level of education, and household wealth all influence the prevalence of polygyny (Okorje, 1994; Strassmann, 1997; CPS/MS, 2007). Poverty and education have a symbiotic relationship. This is because education provides knowledge and skills that lead to higher wages (Cremin & Nakabugo, 2012). When household funds are needed in most rural areas, parents are willing to forego their daughters' education and instead ask them to participate in economic activities to supplement household income (Morley, Leach, & Lugg, 2009; Morley, Lihamba, Mwaipopo, Forde, & Egbenya, 2010). Due to the increasing incidence of poverty in rural Sub-Saharan Africa, girls are vulnerable to very few alternative income opportunities other than inside the bounds of marriage, which can result in very rapid marriage transactions in both families (UNICEF, 2018). These relationships were repeated in this present study; those rural

residents were more likely to engage in polygyny marriage compared to those urban residents. In addition, those without a history of education were more likely to engage in polygyny marriage unions compared to those with.

More so, the southern regions of Ghana are more urbanized than the northern regions of Ghana (GSS, 2014). This explains why those of the northern region were more likely to engage in polygyny marriage union compared to those of the Western region (southern region). This is a further clarification of why women of ethnic groups in northern regions were more likely to engage in polygyny marriage unions when compared to those of the Akan tribe of southern Ghana. Those of the Mole-Dagbani tribe were twice more likely to engage in polygyny marriage unions when compared to those of the Akan tribe. Again, those of the Gurma tribe were almost three times more likely to engage in polygyny marriage unions when compared to those of Akan.

Nevertheless, women with a history of the earlier sexual debut were more likely to engage in polygyny marriage unions compared to those without. In a recent study, the marriage status of women was associated with a history of earlier sexual debut (Alhassan, Abdulai, & Alhassan, 2021). Maybe women with early exposure to sex will prefer polygynous marriage instead of sticky to promiscuous sex. Ghana's male-to-female ratio was 102.79 males per 100 females in 2020, up from 102.62 males per 100 females in 2015, representing a 0.16 percent increase (Index mundi, 2021).

Lastly, women with a history of being in union more than once were more likely to engage in polygyny marriage unions compared to those without. This means that once married, women prefer polygynous marriage to being single and promiscuous sex.

This study is not without limitations as not all factors including economic factors were explored in this study. However, the strength of this study is that the dataset employed is the national presentative survey by demographic and health survey.

Conclusion

The prevalence of polygyny in this present study is high given the strict position the Christian religion has against polygyny. The predictor factors identified include the age of the woman, history of education, type of place of residence, region, ethnicity, early sex initiation, and history of multiple unions.

Data Availability

All dataset related to the findings of this study is available online at www.dhsprogram.com.

Conflict of Interest

There is no conflict of interest with this submission.

Funding Statement

Funding for this study was completed by Ghana Organization for Maternal and Child Health (GOMaCH).

References

Adewale, D., Dey, N. E., Ansah, K. O., Duah, H. O., & Agbadi, P. (2021). attitude towards intimate partner violence against women in Ghana. *Social Sciences & Humanities Open, 4*(1), 1-7. doi:https://doi.org/10.1016/j.ssaho.2021.100207

Ahinkorah, B. (2021). Polygyny and intimate partner violence in sub-Saharan Africa: evidence from 16 cross-sectional demographic and health surveys. *SSM-Population Health, 13*, 100729. doi:https://doi.org/10.1016/j.ssmph.2021.100729

Alhassan, A. R., Abdulai, K., & Alhassan, M. A. (2021). Early Sexual Debut among Ghanaian Women: Correlates and Psychological Effect. *BioMed Research International, 2021*, 9. doi:https://doi.org/10.1155/2021/5838510

Al-Krenawi, A. (2001). Women from polygamous and monogamous marriages in an out-patient psychiatric clinic. *Transcult Psychiatry., 38*(2), 187–99. . doi:https://doi.org/10.1177/136346150103800203

Al-Krenawi, A., & Graham, J. (2009). Helping professional practice with indigenous peoples: the Bedouin-Arab case. *University Press of America*.

Al-Krenawi, A., & Slonim-Nevo, V. (2008). Psychosocial and familial functioning of children from polygynous and monogamous families. *J Soc Psychol., 148*(6), 745–64. doi:https://doi.org/10.3200/SOCP.148.6.745-764

Bove, R., & Valeggia, C. (2009). Polygyny and women's health in sub-Saharan Africa. *Soc Sci Med., 68*(1), 21–9. doi:https://doi.org/10.1016/j.socscimed.2008.09.045

Cellule de Planification et de Statistique du Ministe` re de la Sante´ (CPS/MS). (2007). *Direction Nationale de la Statistique et de l'Informatique du Ministe`re de l'E´conomie, de l'Industrie, et du Commerce (DNSI/MEIC) et Macro International Inc. In: Enqueˆte De´mographique et de Sante´ du Mali 2006.* . Calverton, Maryland, USA: CPS/DNSI et Macro International Inc.

Cremin, P., & Nakabugo, M. G. (2012). Education, development and poverty reduction: A literature critique. *International Journal of Educational Development, 32*(4), 499-506. Retrieved from https://eric.ed.gov/?id=EJ964323

Elbedour, S., Onwuegbuzie, A., Caridine, C., & Abu-Saad, H. (2002). The effect of polygamous marital structure on behavioral, emotional, and academic adjustment in children: a comprehensive review of the literature. *Clin Child Fam Psychol Rev., 5*(4), 255–71. doi:https://doi.org/10.1023/A:1020925123016

Fulas, D. (2018). *The legal framework regulating polygamy in Ethiopia: an assessment in light of Liberal feminist legal theory and international human rights law.*

Gaffney-Rhys, R. (2012). A comparison of child marriage and polygamy from a human rights perspective: are the arguments equally cogent? *J Soc Welfare Fam, 34*(1), 49–61. doi:https://doi.org/10.1080/09649069.2012.675464

Ghana Statistical Service. (2018). *Ghana Maternal Health Survey 2017.* Accra, Ghana: Ghana Health Service and ICF.

Gibson, M., & Mace, R. (2007). Polygyny, reproductive success and child health in rural Ethiopia: why marry a married man? *J Biosoc Sci., 39*(2), 287–300. doi:https://doi.org/10.1017/S0021932006001441

GSS. (2014). *2010 Population & Housing Census Report Urbanization.* Accra: Ghana Statistical Service.

Hadley, C. (2005). Is polygyny a risk factor for poor growth performance among Tanzanian agropastoralists? *Am J Phys Anthropol., 126*(4), 471–80. doi:https://doi.org/10.1002/ajpa.20068

Hassouneh-Phillips, D. (2001). Polygamy and wife abuse: a qualitative study of Muslim women in America. *Health Care Women Int., 22*(8), 735–48. doi:https://doi.org/10.1080/073993301753339951

Holst, R. (1967). POLYGAMY AND THE BIBLE. *International Review of Mission*, 205-213. doi:https://doi.org/10.1111/j.1758-6631.1967.tb03450.x

Index mundi. (2021, September 18). *Ghana Sex ratio*. Retrieved from Index mundi: https://www.indexmundi.com/ghana/sex_ratio.html

Jacoby, H. (1995). The economics of polygyny in sub-Saharan Africa: female productivity and the demand for wives in Côte d'Ivoire. *J Polit Econ., 103*(5), 938–71. doi: https://doi.org/10.1086/262009

Lawson, D., & Gibson, M. (2018). Polygynous marriage and child health in sub-Saharan Africa: what is the evidence for harm? *Demogr Res., 39*, 177–208. doi:https://doi.org/10.4054/DemRes.2018.39.6

Morley, L., Leach, F., & Lugg, R. (2009). Democratising higher education in Ghana and Tanzania: Opportunity structures and social inequalities. *International Journal of Educational Development, 29*, 56-64.

Morley, L., Lihamba, A., Mwaipopo, R., Forde, L. D., & Egbenya, G. (2010). *Widening participation in higher education in Ghana and Tanzania: Developing an equity scorecard (An ESRC/DFID Poverty Reduction Program Research Project,.* Retrieved from http://www.sussex.ac.uk/wphegt

Mwambene, L. (2017). What is the future of polygyny (polygamy) in Africa? *J Potchefstroomse Elektroniese Regsblad., 20*(1), 1–33.

Okorje, A. (1994). African polygamy: church action,· community reaction, and· personal interaction. *Indian J Theol., 36*(2), 2–9.

Omariba, D., & Boyle, M. (2007). Family structure and child mortality in sub-Saharan Africa: cross-national effects of polygyny. *Marriage Fam., 69*(2), 528–43. doi:https://doi.org/10.1111/j.1741-3737.2007.00381.x

Slonim-nevo, V., & Al-Krenawi, A. (2006). Success and failure among polygamous families: the experience of wives, husbands, and children. *Fam Process, 45*(3), 311–30. doi:https://doi.org/10.1111/j.1545-5300.2006.00173.x

Smith-Greenaway, E., & Trinitapoli, J. (2014). Polygynous contexts, family structure, and infant mortality in sub-Saharan Africa. *Demography, 51*(2), 341–66. doi:https://doi.org/10.1007/s13524-013-0262-9

Strassmann, B. (1997). Polygyny as a risk factor for child mortality among the Dogon. *Curr Anthropol, 38*(4), 688–95. doi:https://doi.org/10.1086/204657

SWAC/OECD. (2019, 01 18). Polygamy remains common and mostly legal in West Africa. *West Africa Brief*, pp. 1-2. Retrieved from http://www.west-africa-brief.org/content/en/polygamy-remains-common-and-mostly-legal-west-africa

Thobejane, T., & Flora, T. (2014). An exploration of polygamous marriages: A worldview. *Mediterr J Soc Sci., 5*(27), 1058.

Trócaire. (2017). *"Understanding women's lives in polygamous marriages: exploring community perspectives in DRC and Sierra Leone.* Trócaire;.

UNICEF. (2018). *Child Marriage:Latest trends and future prospects.* New York: UNICEF.

Figure legends

Figure 1: Prevalence of Christian women involved in a polygyny marriage

Figure 2: The rank of Christian women involved in a polygyny marriage

Table 1: Demographic characteristics of study participant

		Frequency	Percentage
Age of respondent	15-19	105	1.6%
	20-24	531	8.3%
	25-29	1134	17.7%
	30 and above	4623	72.3%
Ever attended school	Yes	4486	70.2%
	No	1907	29.8%
Type of place of residence	Urban	2962	46.3%
	Rural	3431	53.7%
Christian denomination	Catholic	1419	22.2%
	Anglican	61	1.0%
	Methodist	336	5.3%
	Presbyterian	367	5.7%
	Pentecostal/charismatic	3139	49.1%
	Other Christian	1071	16.8%
Region	Western	697	10.9%
	Central	414	6.5%
	Greater accra	667	10.4%
	Volta	282	4.4%
	Eastern	667	10.4%
	Ashanti	770	12.0%
	Brong ahafo	544	8.5%
	Northern	689	10.8%
	Upper east	1046	16.4%
	Upper west	617	9.7%
Ethnicity	Akan	2557	40.0%
	Ga/dangme	303	4.7%
	Ewe	656	10.3%
	Guan	157	2.5%
	Mole-dagbani	1856	29.0%
	Grusi	267	4.2%
	Gurma	532	8.3%
	Mande	27	0.4%
	Other	38	0.6%
Child marriage	No	4496	70.3%
	Yes	1897	29.7%
Early sex initiation	No	3169	49.6%
	Yes	3220	50.4%

In union more than once	Only once	5445	85.2%
	More than once	948	14.8%

Table 2: Chi-square analysis of factors associated with polygyny marriage relations among Christian women of Ghana

		In Polygyny marriage		Test statistics	
		No	Yes		
Age of respondent	15-19	97	6	Chi-square	67.881
	20-24	490	39	Sig.	.000*
	25-29	1053	74		
	30 and above	3945	659		
Ever attended school	Yes	4161	299	Chi-square	423.851
	No	1424	479	Sig.	.000*
Type of place of residence	Urban	2799	144	Chi-square	274.426
	Rural	2786	634	Sig.	.000*
Region	Western	638	54	Chi-square	340.409
	Central	377	34	Sig.	.000*
	Greater Accra	641	23		
	Volta	239	42		
	Eastern	632	33		
	Ashanti	726	38		
	Brong Ahafo	482	58		
	Northern	505	183		
	Upper east	849	193		
	Upper west	496	120		
Ethnicity	Akan	2417	126	Chi-square	410.767
	Ga/Dangme	286	15	Sig.	.000*
	Ewe	587	64		
	Guan	144	12		
	Mole-Dagbani	1481	369		
	Grusi	241	26		
	Gurma	370	160		
	Mande	25	2		
	Other	34	4		
Child marriage	No	4022	449	Chi-square	66.857
	Yes	1563	329	Sig.	.000*
Early sex initiation	No	2889	265	Chi-square	85.604
	Yes	2692	513	Sig.	.000*
In union more than once	Only once	4805	613	Chi-square	28.323
	More than once	780	165	Sig.	.000*

Table 3: Binary logistics analysis of predictor factors of polygyny marriage relations among Christian women of Ghana

		B	Sig.	AOR	95% C.I. for AOR	
					Lower	Upper
Age of respondent	15-19	Ref				
	20-24	.597	.195	1.816	.737	4.474
	25-29	.763	.088	2.145	.894	5.148
	30 and above	1.849	≤ 0.001	6.354	2.713	14.883
Ever attended school	Yes	Ref				
	No	.511	≤ 0.001	1.666	1.365	2.033
Type of place of residence	Urban	Ref				
	Rural	.769	≤ 0.001	2.157	1.728	2.692
Christian denomination	Catholic	Ref				
	Anglican	.349	.397	1.418	.632	3.182
	Methodist	.616	.011	1.851	1.152	2.972
	Presbyterian	.536	.010	1.709	1.135	2.574
	Pentecostal/charismatic	.367	≤ 0.001	1.444	1.159	1.800
	Other Christian	.180	.209	1.197	.904	1.584
Region	Western	Ref				
	Central	.303	.204	1.354	.849	2.161
	Greater Accra	-.243	.387	.784	.452	1.361
	Volta	.426	.115	1.531	.901	2.600
	Eastern	-.435	.073	.647	.402	1.041
	Ashanti	-.310	.173	.733	.469	1.146
	Brong Ahafo	-.076	.728	.927	.605	1.420
	Northern	.538	.012	1.712	1.128	2.598
	Upper east	.358	.092	1.430	.943	2.169
	Upper west	.232	.307	1.261	.808	1.967
Ethnicity	Akan	Ref				
	Ga/Dangme	.229	.451	1.258	.693	2.281
	Ewe	.527	.010	1.693	1.134	2.530
	Guan	.072	.830	1.075	.557	2.075
	Mole-Dagbani	1.089	≤ 0.001	2.971	2.072	4.260
	Grusi	.434	.109	1.544	.907	2.626
	Gurma	1.313	≤ 0.001	3.719	2.495	5.542
	Mande	.008	.991	1.008	.225	4.520
	Other	.809	.156	2.245	.734	6.865
Child marriage	No	Ref				
	Yes	.098	.317	1.103	.911	1.335
Early sex initiation	No	Ref				

	Yes	.389	≤ 0.001	1.476	1.217	1.789
In union more than once	Only once	Ref				
	More than once	.583	≤ 0.001	1.792	1.451	2.213

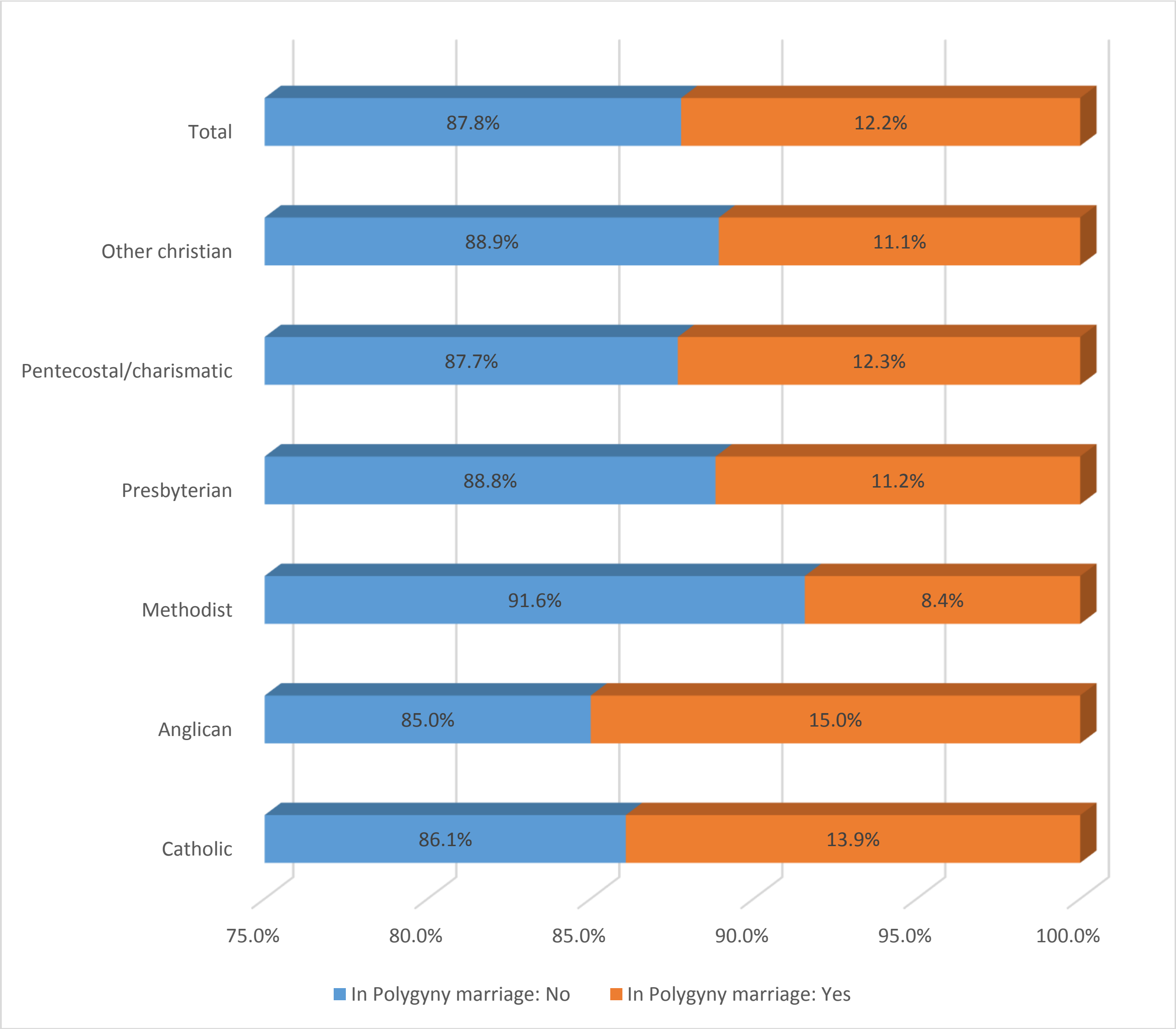

$\boldsymbol{X^2}$**=10.412, P=0.064**

Figure 1: Prevalence of Christian women involved in a polygyny marriage

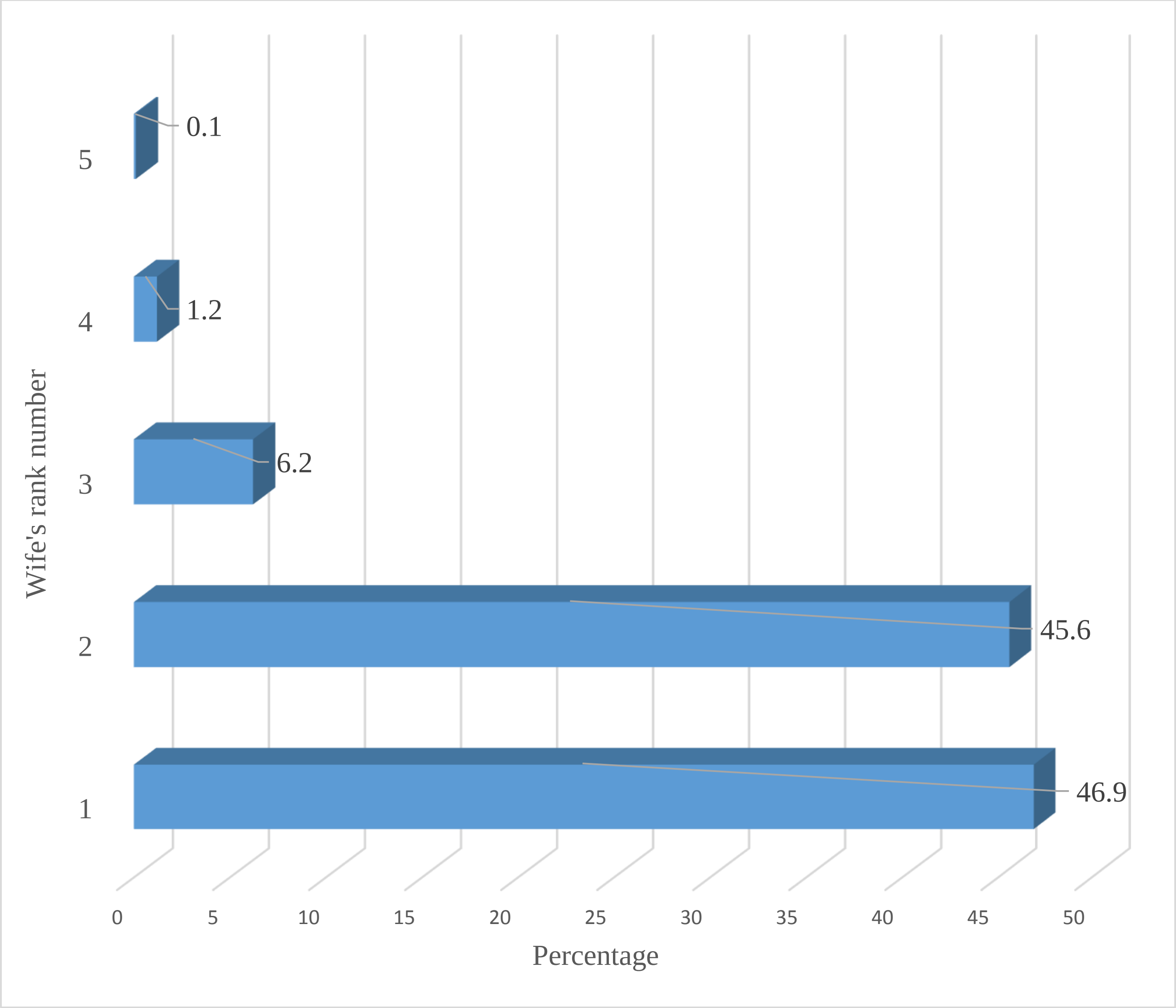

Figure 2: The rank of Christian women involved in a polygyny marriage
