# Supplementary material for: Polygynous marriage union among Ghanaian Christian women: Socio-demographic predictors
Source: PLoS One. 2023 Apr 27;18(4):e0275764. doi: 10.1371/journal.pone.0275764 (PMC10138464; doi:10.1371/journal.pone.0275764)
Supplement: Attachment — Submitted filename: Comments-PONE.docx [file pone.0275764.s001.docx]

Reviewer’s comments

**Abstract**

The abstract is a true reflection of the overall study as presented.

**Introduction**

Literature review is satisfactory and is focused and aligned with the topic and objective.

The justification for the study was satisfactory.

**Methodology**

The authors should have given a brief socio-demographic description of Ghana, since it is national study, highlighting religious background of the country.

Ethical considerations for the study was adequate.

**Results**

The analysis and findings are in tandem with objective of the study.

However, the variables in the tables should be placed directly above the subcategories, to enhance readability. For instance.

| **Factors associated with polygyny** |  |
| --- | --- |
| **Age of respondent** |  |
| 15-19 |  |
| 20-24 |  |
| 25-29 |  |

The presentation of the results is not satisfactory; the percentages should be included in table 2.

**Discussions/Conclusions**

Page 11 paragrah 2, was repeated verbatim in page 15 paragraph 2.

Since the study population were Christians, the authors should have included comparison with findings among Christians elsewhere or other religious groups in addition to Ghana’s general population.

Otherwise, the discussion was satisfactory and was based on the findings from the analysis.

**References**

Most of the references are relevant and up to date.
