# Supplementary material for: Polygynous marriage union among Ghanaian Christian women: Socio-demographic predictors
Source: PLoS One. 2023 Apr 27;18(4):e0275764. doi: 10.1371/journal.pone.0275764 (PMC10138464; doi:10.1371/journal.pone.0275764)
Supplement: Attachment — Submitted filename: Response to reviewer.docx [file pone.0275764.s003.docx]

**Academic editor comments**

**Comment:**

The references should be reviewed to at least 70% of references not older five years.

**Action:**

The references were reviewed to at least 70% of references not older five years.

**Comment:**

There many grammatical errors in the article.

**Actions:**

Grammatical errors in the article attended to.

**Reviewer’s comments**

**Comment (Methodology):**

Highlight religious background of Ghana

**Actions:**

Religious background of Ghana highlighted under Materials and Methods.

**Comment (Results):**

Reformat tables

**Actions:**

**Comment (Discussion):**

1. Sentence copied verbatim from on paragraph to the other.
2. Include comparison with Christian study somewhere.

**Actions:**

1. Verbatim copied sentences edited
2. Christian studies somewhere compared in the discussion.
