# Supplementary material for: Polygynous marriage union among Ghanaian Christian women: Socio-demographic predictors
Source: PLoS One. 2023 Apr 27;18(4):e0275764. doi: 10.1371/journal.pone.0275764 (PMC10138464; doi:10.1371/journal.pone.0275764)
Supplement: Attachment — Submitted filename: Response to reviewer- B.docx [file pone.0275764.s004.docx]

**Editor’s comments**

**Comments**: Your method of referencing did not adhere to that recommended by PLOS journals, and in addition, they are not numbered. Please read the method of referencing recommended by the PLoS One journal on the relevant page on our website, and make the appropriate corrections.

**Author’s Response**

**Responses**: The referencing styles updated to Vancouver using Microsoft office referencing.
